# 

**Published:** 1906-01

**Authors:** 


### BOOKS RECEIVED.

A Manual of the Practice of Medicine. By A. A. Stevens, A.M., M.D., Professor of Pathology in the Woman's Medical College of Pennsylvania, and Lecturer on Physical Diagnosis at the University of Pennsylvania. Seventh edition, revised. 12mo of 556 pages, illustrated. Philadelphia and London: W. B. Saunders & Co. 1905. Flexible leather, $2.50 net.)

Gray's Anatomy. Descriptive and Surgical. New American from the 15th English edition. Revised, enlarged and rewritten by J. Chalmers DaCosta, M.D., Professor of Surgery in the Jefferson Medical College, in collaboration with a corps of specially selected assistants. In one very handsome imperial octavo volume of 1600 pages, with 1132 illustrations, 500 of which are new in this edition. (Price, with illustrations in black: cloth, $5.50 net; leather, $6.50 net.

Price, with illustrations in black and many colors: cloth, $6.00 net; leather, $7.00 net.) Lea Brothers & Co., Publishers, Philadelphia and New York. 1905.

Progressive Medicine, Vol. IV, December, 1905. A quarterly digest of Advances, Discoveries and Improvements in the Medical and Surgical Sciences. Edited by Hobart Amory Hare, M.D., Professor of Theraputics and Materia Medica in the Jefferson Medical College of Philadelphia. Octavo, 367 pages, 41 engravings, and 5 full-page colored plates. Per annum, in four cloth-bound volumes, $9.00; in paper binding, $6.00; carriage paid to any address. Lea Brothers & Co., Publishers, Philadelphia and New York.

Le Fevre's Diagnosis. A Manual of Physical Diagnosis, including Diseases of the Thoracic and Abdominal organs. For students and Physicians. By Egbert Le Fevre, M.D., Professor of Clinical Medicine and Theraputics in the University and Bellevue Hospital Medical College, Attending Physician to Bellevue Hospital and to St. Luke's Hospital, New York. New (2d) edition, thoroughly revised and much enlarged. In one 12mo volume of 479 pages with 102 engravings and 6 full page plates in black and colors. Cloth, $2.25, net. Lea Brothers & Co., Publishers, Philadelphia and New York.

Lectures on Auto-Intoxication in Disease, or Self-Poisoning of the Individual. By Charles Bouchard, Professor of Pathology and Theraputics; Member of the Academy of Medicine and Physician to the Hospitals, Paris. Translated, with a Preface and New Chapters added, by Thomas Oliver, M.A., M.D., F.R.C.P., Professor of Physiology, University of Durham; Physician to the Royal Infirmary, New Castle-Upon-Tyne; Formerly Examiner in Medicine, Royal College of Physicians, London. Second revised edition. Crown Octavo, 342 pages, Extra Cloth. Price, $2.00, net. F. A. Davis Company, Publishers, 1914-16 Cherry Street, Philadelphia.

Minor and Operative Surgery, including Bandaging. By Henry R. Wharton, M.D., Profesor of Clinical Surgery in the Woman's College; Surgeon to the Presbyterian Hospital, Philadelphia, etc. New (6th) edition, enlarged and thoroughly revised. In one 12mo. volume of 642 pages, with 532 illustrations. Cloth, $3.00, net. Lea Brothers & Co. Publishers, Philadelphia and New York, 1905.

Berg's Surgical Diagnosis. A manual of Surgical Diagnosis. For Students and Practitioners. By Albert A. Berg, M.D., Adjunct Attending Surgeon to Mt. Sinai Hospital, New York. In one 12mo. volume of 543 pages with 215 engravings and 21 full page plates. Cloth, $3.25, net. Lea Brothers & Co., Publishers, Philadelphia and New York.

A Textbook of Modern Materia Medica and Theraputics. By A. A. Stevens, A.M., M.D., Lecturer on Physical Diagnosis, University of Pennsylvania; Professor of Pathology, Woman's Medical College of Philadelphia. Fourth edition, revised. Octavo of 670 pages. Philadelphia and London: W. B. Saunders & Company, 1905. Cloth, $3.50 net.

Urinary Analysis and Diagnosis by Microscopical and Chemical Examination. By Louis Hertzmann, M.D., of New York. Second edition, revised and enlarged, with 131 illustrations (mostly original). New York: William Wood and Company 1906. Octavo pp. 319. (Price, $2.00, Cloth.)

Differential Diagnosis and Treatment of Disease. A textbook for Practitioners and Advanced Students, by Augustus Caillé, M.D.,

Professor of Diseases of Children, New York Post-Graduate Medical School and Hospital, etc. With 228 illustrations in the text. Octavo pp. 867. New York and London: D. Appleton and Company. 1906. (Price, Cloth $6.00 net.)

Pharmacology and Theraputics. By Reynold Webb Wilcox, M.A., M.D., LL.D., Professor of Medicine at the New York Post-Graduate Medical School and Hospital, and Attending Physician to the Hospital; Consulting Physician to the Nassau Hospital, etc. Small octavo pp. 1010. Sixth edition. Philadelphia: P. Blakiston's Son & Co. 1905. (Price, Cloth $3.00 net.)

Blakiston's Quiz Compends. A Compend of Medical Chemistry, Inorganic and Organic, including Urinary Analysis. By Henry Leffman, A.M., M.D., Professor of Chemistry in the Woman's Medical College. Fifth edition, revised. Philadelphia: P. Blakiston's Son & Co. 1905. (Price, $1.00.)

Man and His Poisons. A practical Exposition of the Causes, Symptoms and Treatment of Self-Poisoning. By Albert Abrams, A.M., M.D., Consulting Physician to the Denver National Hospital for Consumptives. Illustrated, pp. 268. New York: E. B. Treat & Company. 1906. (Price, 1.50 net.)

Williams on Food. Food and Diet in Health and Disease. A Manual for Practitioners of Medicine, Students, Nurses and the Lay Reader. By Robert F. Williams, Professor of Principles and Practice of Medicine in the Medical College of Virginia, Richmond. In one handsome 12mo volume of 392 pages. Cloth, net, $2.00. Lea Brothers & Co., Publishers, Philadelphia and New York, 1906.

Gallstones and Their Surgical Treatment. By B.. G. A. Moynihan, M.S. (London), F.R.C.S., Senior Assistant Surgeon to Leeds General Infirmary, Leeds, England. Second edition, revised and enlarged. Octavo of 458 pages, beautifully illustrated. Philadelphia and London: W. B. Saunders & Company, 1905. (Cloth, $5.00 net; Half Morocco, $6.00 net.)

Dosebook and Manual of Prescription Writing: with a list of the Official Drugs and Preparations, and the more important Newer Remedies. By E. Q. Thornton, M.D., Asistant Professor of Materia Medica, Jefferson Medical College, Philadelphia. Third Edition, Revised and Enlarged. 12mo, 392 pages, illustrated. Philadelphia and London: W. B. Saunders & Company, 1905. Bound in flexible leather, $2.00 net.

Saunders's Question Compends. Essentials of Materia Medica, Therapeutics, and Prescription Writing. By Henry Morris, M.D., College of Physicians, Philadelphia. Seventh edition, thoroughly revised. By W. A. Bastedo, Ph.G., M.D., Instructor in Materia Medica and Pharmacology at the Columbia University (College of Physicians and Surgeons), New York City. 12mo, 300 pages. Philadelphia and London: W. B. Saunders & Company, 1905. (Cloth, $1.00 net.)

Nervous and Mental Diseases. By Archibald Church, M.D., Professor of Nervous and Mental Diseases and Medical Jurisprudence in Northwestern University Medical School, Chicago; and Frederick Peterson, M.D., President of the State Commission in Lunacy, New York; Clinical Professor of Neurology and Psychiatry, Columbia University. Fifth edition, revised and enlarged. Octavo volume of

937 pages, with 341 illustrations. Philadelphia and London: W. B. Saunders & Co., 1905. (Cloth, $5.00, Half Morocco, $6.00, net.)

Culbreth's Materia Medica. A Manual of Materia Medica and Pharmacology for Students and Practitioners of Medicine and Pharmacy. Comprising all Organic and Inorganic Drugs which are and have been official in the United States Pharmacopeia, together with important Allied Species and Useful Synthetics. By David M. R. Culbreth, Ph.G., M.D., Professor of Botany, Materia Medica and Pharmacology in the University of Maryland, Departments of Medicine, Pharmacy and Dentistry. Fourth edition. Revised to accord with the new U. S. Pharmacopeia, 8th Decennial Revision. Octavo, 976 pages, 487 illustrations. Lea Brothers & Co., Publishers, Philadelphia and New York. 1906. (Cloth, $4.75, net.)

Cushny's Pharmacology. A textbook of Pharmacology and Theraputics: The Action of Drugs in Health and Disease. By Arthur R. Cushny, M.A., M.D., Aberd., Professor of Pharmacology in the University College, London; formerly Professor of Materia Medica and Theraputics in the University of Michigan. In one handsome octavo volume of 752 pages, with 52 illustrations. Lea Brothers & Co., Publishers, Philadelphia and New York. 1906. (Cloth $3.75 net).

A Manual of Bacteriology. By Herbert U. Williams, M.D., Professor of Pathology and Bacteriology in the Medical Department of the University of Buffalo. Revised by B. Meade Bolton, expert in the Bureau of Animal Industry, Washington, D.C. With 108 illustrations. Fourth edition, revised and enlarged. Philadelphia: P. Blakiston's Son & Co. 1905. (Price, Cloth, $1.50.)

